# Abstracts and Gleanings

**Published:** 1887-05

**Authors:** 


### ABSTRACTS AND GLEANINGS.

**The Treatment of Pneumonia.**—The *Medical News* remarks: There is a tendency at present to revive venesection, and it is important to understand clearly the indications for its practice. Unquestionably prompt and free bleeding is called for in threatened death from asphyxia when the right heart is distended, cyanosis marked, and the pulse becoming small and rapid. The timely abstraction of sixteen to twenty ounces of blood will sometimes save life, though we must confess to some disappointment in the results, for of five such cases observed, only one recovered. The practice of free bleeding at the onset, which is still employed by some practitioners, is not to be commended, except in rare cases.

In spite of the fact that the leading text books on Practice in this country, and many of the prominent teachers discourage the use of arterial sedatives in pneumonia, they are still most extensively employed. It is true that tartar emetic has lost favor, but aconite and veratrum viride are still favorite remedies, and there are practitioners who claim wonderful results from their use. We have never been able to satisfy ourselves that they have the slightest influence upon the duration of the disease, and on the other hand, they tend to depress the heart, and may thus be directly injurious if continued for many days. The calomel treatment has been revived of late, but the careful reports from Botkin's clinic, in St. Petersburg, do not indicate that it has any value in controlling the severity of the disease.

Opium in the form of Dover's powder is a most valuable remedy in the early stage of the disease, and was much used in Edinburgh some years ago. It is contra-indicated if there is any tendency to accumulation in the bronchial tubes, but in the painful onset it is often of signal service in allaying the pain and restlessness. We doubt if it has had a sufficient trial in the hands of practitioners.

While many cases of pneumonia do well if carefully nursed and fed, there arise in others two indications demanding the most judicious treatment—viz: high fever and heart failure. So long as the temperature does not rise above 103°, the use of antipyretics is not necessary; but, with a temperature above this, quinine should be given in from 20 to 25 grain doses. Antipyrin is not so suitable, as it is decidedly more depressing than the quinine, and the same objection holds with thalin and kairin. Repeated cold sponging is a useful adjunct, and in the combination of high temperature with delirium and great restlessness the cold pack is indicated. In the heart failure of which so many pneumonic patients die there is as yet no substitute for alcohol, which, as in typhoid fever, is well borne. Camphor, as a cardiac stimulant, has valuable uses.

A study of the history of the treatment of pneumonia makes one almost despair of the future of therapeutics, so impossible does it seem to arrive at reliable conclusions regarding the use of medicines. And yet our feelings of despair vanish when we compare the figures of to-day, bad as they appear, with those of the first quarter of this century; for we then see that there has been in the treatment of pneumonia. as in that of fevers, a steady, progressive enlightenment. The heavy mortality is due largely to the fact that pneumonia attacks with special avidity the aged, the weak, and the dissipated.

**Diphtheria**.—Dr. Patterson (in *N. C. Medical Journal*) says: In a prize essay by the late Dr. E. S. Gaillard, and published in the *Richmond Medical Journal* in 1886, he recommended as a chief reliance in its treatment the following prescription:

R. Dilute muriatic acid..............................ʒ ij,

Tinct. muriate iron..............................ʒ ij,

Chlorate potash..............................ʒ i,

Water.......................................℥ xii.

Of this 2 tablespoonfuls are to be given every hour through the day and twice during the night to the adult; less to children according to age.

In a pamphlet issued by the Rio Chemical Company, of St. Louis, Dr. Cool recommended for diphtheria an application to the parts covered by the membrane by means of a mop or probang a mixture of Kennedy's pinus canadensis, 1 ounce, and carbolic acid, 10 drops. Combining these two remedies, as soon as the patient is seen I commence and endeavor to obtain the constitutional effects of the solution as soon as possible, immediately after each dose of which the mopping is done with the pinus. The membrane turns black and in a few days, at farthest, drops off, not to return again. Amelioration of all the symptoms takes place and convalescence is soon established. At least, that was the result in the twenty cases referred to above without an exception. It seems to arrest the disease before it has time to extend to the air passages, from which cause it is estimated that three-fourths of the deaths result. Constipation was removed by enema—cathartics seldom necessary. Debility treated by alcoholic drinks, milk, eggs, quinine.

**Treatment of Chorea**.—In a recent number of the *Medical and Surgical Reporter*, Dr. Hiram Corson emphatically calls attention to the value of cimicifuga racemosa in chorea of childhood. He affirms, as the result of fifty years of experience, that it is always successful in a brief time if a teaspoonful of a good fluid extract be given four times a day. This use of cimicifuga racemosa is a very old one, which was insisted upon by the late Dr. George B. Wood, and which, as pupils of that great master, we have long employed.

Some hundreds of cases of chorea have come under our care in the public service at the Philadelphia Hospital, and especially at the University Hospital. In the earlier years the fluid extract of cimicifuga racemosa was always relied upon and administered as soon as the patients presented themselves. Experience has emphatically taught us, however, that it is distinctly inferior to arsenic; so that at present every patient coming to the Dispensary with the St. Vitus' dance is put upon the arsenical treatment. In the few cases in which this fails, the next routine administration is of the fluid extract of cimicifugia. We can only explain the superiority which cimicifuga has asserted over arsenic in the hands of Dr. Corson by the supposition that the doctor has never used arsenic with sufficient freedom.

The arsenic preparation must be given in ascending doses until it produces evidences of its physiological action, and to order this requires a little boldness on the part of the physician. If, however, the patient be well watched and the remedy be withdrawn as soon as puffiness appears in the face, no harm can be done. Cimicifuga is not an inert substance, as seems to be thought by some practitioners. Probably much of the cimicifuga that is administered has lost its activity, which appears to depend upon a volatile principle. But we have seen a teaspoonful of the good fluid extract, even in an adult, produce headache, with excessive giddiness and great prostration.—*Therapeutic Gazette.*

**Arseniate of Strychnine.**—It is claimed that the drug has given excellent remedial results, and that it acts not only as arsenic but also as strychnine. The preparation, which is soluble in water and glycerin in every proportion, is well suitable for hypodermic medication, produces no pain, and is easily absorbable.

Nevertheless, Russel recommends to use in the beginning only small doses, as a dose containing 1/6 of a grain of arseniate of strychine produces toxic symptoms when exhibited in the beginning of the treatment. The author himself employs usually a solution of 1 to 250. He begins with 1/4 to 1/2 of a syringe, and gradually arrives at the full syringe, containing 1-16 of a grain.

Russel states that the effect of the foregoing full dose is peculiarly striking in the premonitory symptoms of the acute infectious diseases, such as malaise, depression, muscular lassitude, etc., the patient feeling revived, as it were, after a single injection. This singular effect of the drug has been so constantly observed by the author in the above conditions that he employs it systematically in all affections marked by a great reduction of vital energy.

The main importance, however, of the new remedy consists in another and entirely different feature, *i. e.*, in its powerful antiseptic properties, which raise the drug to an agent of the highest rank and service in typhoid fever. Such, at least, is the conviction and the claim of Dr. Russel, who, within the last four years, has repeatedly succeeded by means of this drug to either abort an incipient typhoid fever, or to supplant the morbid process when already fully developed.

The following case, reported by Dr Russel in this connection, invites our interest. Two sisters were attacked simultaneously by typhoid fever (ileotyphus), and for various reasons one of them alone was given into his care. The doctor injected a Pravaz syringful of the arseniate of strychnine, and had the satisfaction of finding his patient after eight days convalescing from a light case of abortive typhoid fever. The other sister was presented for treatment at the tenth day of sickness, when the patient had already entered the second period of the affection. Having instituted here likewise the treatment with arseniate of strychnine, the course of the affection was rendered simple, and recovery soon ensued.

It seems, then, as if the new drug is endowed with great disinfecting powers, capable of sterilizing, as it were, the soil on which the pathogenetic micro-organisms of typhoid fever vegetate.

In addition to this special indication, Russel recommends the drug also in combination with salicylate of iron in chronic anæmia and dyspepsia, and states that he has repeatedly tested the efficacy of the drug in these affections.—*Therapeutic Gazette.*

**A New Method of Dilating the Uterus.**—Dr. Vulliet, professor in the Medical Faculty at Geneva, communicated at the meeting on April 6, 1886, of the Academy of Medicine in Paris, his new method of dilating the uterus, and its application in the diagnosis and treatment of uterine disorders. The following is a summary of the commmunication. His method (1) renders possible the direct inspection of the whole uterine cavity; (2) permits the dilatation to be continued, whatever be its degree, as long as desirable and necessary; (3) affords at the same time the most efficient antiseptic dressing. Professor Vulliet dilates the uterus by plugging the cavity with iodoform tampons, which he leaves. Eight to ten pluggings, repeated every two to eight hours with gradually enlarged tampons, will on the average suffice to render visible the cavity, the patient being in the genu-pectoral position. A dilatation of sufficient degree as to open to view the whole interior, facilitates the diagnosis and treatment of all affections of the cavity. Professor Vulliet has employed it in the treatment of cancer, of submucous and parietal fibro-myomata, and of chronic endometritis. He has obtained, within eight months, complete cicatrization in four cases of uterine cancer, and is hopeful about these local cures. Time, however, must prove whether they be permanent. In parietal fibro-myomata, this method, without endangering the layer of tissue which separates the tumor from the cavity, permitted incisions to be made in order to transform the parietal neoplasm into an easily removable polypus of the cavity. In endometritis, the dilatation facilitates the scraping of the mucous membrane by means of the curette, and a consequent energetic typical treatment. Without pushing dilatation to its extreme possible extent, Vulliet's method will prove very useful on account of the long duration of the dilatatation, and that especially in strictures and deviation of the cervical canal, and in all operations which require the repeated and frequent introduction of the finger or of larger instruments. Moreover, this method—by its very principle—the plugging with antiseptic subtances—is applicable in every case in which virulent processes are evolved in the uterus, namely, puerperal, catarrhal, and blennorhagic infections. Prof. Vulliet, requested by M. Leon Labbe to demonstrate his method in the Hospital Beaujon, succeeded within twenty-four hours in dilating the uterus sufficiently to permit full inspection of the fundus. He has since frequently operated after his method for cancer of the uterus in private practice, and, by invitation, in several hospitals.—*London Medical Record.*

**The Constipation Habit.**—The following I give as a general outline of the treatment, which, of course, must be varied somewhat according to the special indications of each case: Regulate the diet, having three meals per day of palatable, nutritious food, not too bulky or too concentrated. Have soup, at least one meal each day. On rising, at least an hour before breakfast, drink one or two large goblets of water. If the stomach is weak and inclined to chronic gastritis, I order the water to be drank hot. Twenty or thirty minutes following the water, give the bowels a thorough kneading for ten minutes. Then assume erect position, with arms above the head and left foot on a line with the right and placed in front of it, bend forward until the knuckles of the closed hands touch the floor, then back to the first position, repeating this five or six times; then, reversing the position of the feet, repeat the movements. This is an excellent exercise for the abdominal muscles and an inactive liver.

At night also, before retiring, drink a goblet of water, and if there are indications of dryness of lower bowels I use an enemata of one-third to one-half cup of water to be retained. Flushing the sewer may be a good practice with some, making the stomach the flooding-tank; but we must use great care not to interfere with digestion.

When it is available, I often order a fifteen minutes' daily applications of electricity to the abdomen, using the Faradic current.

If any medicine is demanded, the first on the list is cascara sagrada. I think it is an excellent "peristaltic persuader.' It renders in my hands the most efficient service in small and repeated doses.

I impress it upon my patients to make it a daily practice to go to stool at a regular hour, to induce if possible, by voluntary muscular effort, a movement, remembering that this measure alone, if pesisted in, will oftentimes overcome this deplorable habit. Perhaps the best time of the day for this is soon after breakfast. Patient continuation in this line of treatment will do a great deal to dispel this *bete noire* of medical practice.—*Investigator.*

**The Management of Incomplete Abortion.**—Dr. W. A. Edis contributes to the *British Medicat Journal*, a valuable paper on the management of those cases of incomplete abortion where the ovisac has been ruptured, the embryo been expelled in part—the placenta, either entire or in fragments, being retained in utero, and giving rise to hemorrhage or septicæmia in consequence.

The principle of early and complete evacuation of the contents of the uterus, when the vitality of the ovum has been destroyed, although generally accepted by every one competent to give an opinion, is yet too often neglected in practice, and leads to the most calamitous results.

Too much stress cannot be laid upon the absolute necessity of removing all placental *debris* in cases of incomplete abortion.

In the nulliparæ, when every symptom indicates that abortion is inevitable, the object should be to favor the expulsion of the ovum as quickly as possible The best means then at our command is the carbolized sponge-tent, for this not only checks any existing hemorrhage, but also serves to dilate the cervix and facilitate the expulsion of the oqum entire. In these cases when the cervix is undilated and ergot has been too freely administered, the ovisac becomes ruptured, the embryo expelled, but placenta remains behind.

He emphasizes the statement that the mere plugging of the vagina with the view to arresting hemorrhage from the uterus is unscientific and unsatisfactory, and should never be resorted to unless in severe emergencies where the practitioner has not the instruments at hand, or dos not possess the requisite skill for passing the sponge-tent into the cervix uteri. If the tampon has to be used, small pieces of carbolized sponge are far preferable to cotton wool or strips of linen generally used. If a sponge tent is inserted it should not be allowed to remain in the cervix longer than twelve hours, and when removed iododized or carbolized water should be freely injected into the vagina before proceeding further with the manipulations.

If the efforts to remove all particles of the placenta with the fingers should be unavailing, the curette may be employed.

His custom is not to give ergot if only a portion of the ovum has been expelled. The exception is when hemorrhage is severe. —*Practice.*

**Overdoses of Cocaine.**—Cases are now rather frequently reported of serious symptoms, and even death, from cocaine being used in too large doses. In some instances the dose, or rather strength of preparation, was extravagantly high; therefore, the fault lies with the operator rather than the drug.

Dr. Wyette remarked that he had injected as much as three or four grains of the four-per cent. solution at a single operation. It should be employed with greater care in the region of the fifth nerve It was safe to use a larger quantity when the limb was rendered bloodless, as after the operation it could be "milked" out before entering the general circulation, and the remaining quantity be further diminished by the oozing which would take place on temporarily removing the Esmarch bandage.—*N. Y. Journal.*

One of such cases is the following, reported at the New York Pathological Society:

Dr. Robert Newman presented a tumor remooved from the roof of the mouth of a woman fifty-one years old. It had grown gradually during the last twenty years; more rapidly during the last five months. It was so hard that he was unable to remove it with Jarvis' snare, and used an ecraseur. By the aid of cocaine, the operation was painless, and there were no unpleasant after-effects. Only last week he had a case in which injection of twenty-five minims of a four-per cent. solution of cocaine into the bladder was suddenly followed by an intense pain in the left temple, extending around the top of the head, with a feeling as if the whole top of the head had been blown off; the pain had lasted for ten days, with exacerbations. The wound healed kindly in the present case, and was covered with mucuous membrane.—*N. Y. Medical Record.*

**Salt Baths in Fevers.**—Dr. Vavinovitch has made a number of experiments in cases of typhoid fever to determine the relative value of salt and fresh water baths, and formulates his conclusions as follows (*Meditsinskoye Obozreniye*, No. 14, 1886):

1. Salt baths reduce the temperature several tenths of a degree Centigrade more than ordinary baths.
2. The difference in the abstraction of heat is greater the first half hour, gradually becomes less, and is hardly noticeable at the end of three hours
3. Evening baths reduce the temperature in a greater degree than do those in the morning.
4. Salt baths retard the pulse more than ordinary baths.
5. They also reduce the number of respirations per minute, rendering the inspiratory efforts deeper and more prolonged.
6. Salt baths increase the muscular strength in a greater degree than do fresh-water baths.
7. The patients feel better while in the salt bath and after its termination than they do when a fresh bath is given.
8. The difference between salt and fresh baths, as regards the slowing of the pulse and respiration, is not so great as to indicate any very powerful functional activity of the heart and lungs.—*N. Y. Medical Record.*

**Nitro-Glycerin.**—In sea-sickness, reflex vomiting, gastralgia, hepatic colic, tetanus, and hydrophobia the use of nitro-glycerin has been advised by Bartholow, and he adds that it ought to be certainly tried in whooping cough and laryngismus stridulus.

Dr. Burroughs, in the *Therapeutic Gazette*, recommends nitro-glycerine as a substitute for alcohol and other stimulants. He considers 1 or 2 drops of a 1-per cent. solution equal to one ounce of brandy, and has used the drug successfully in opium-poisoning, typhus fever, and other diseases.—*Practice.*

**For the relief** of dental neuralgia and severe headaches due to pregnancy, Prof. J. S. Wellford, of Richmond, has found the following prescription one of the most pleasant and efficacious remedies:

℞. Ferri pyrophos..................................℈ ij,

Spt. ammon. aromat...............................ʒ ij,

Acid citric.......................................℈ i,

Syr. Simplic......................................ʒ vi,

Aq. distiil, q. s. ad.............................℥ iv.

In severe cases dilute phosphoric acid, f ʒ ij may be substituted for the citric acid.—*Practice.*

**Action of Caffein and Theine.**—Leven, in 1868, showed that theine produced convulsions in frogs, while caffeine did not; and that the lethal dose of theine was larger than that of caffeine. This is confirmed by the experiments on frogs, made by Dr. Thos J. Mays, from which the following conclusions are drawn:

Theine and caffeine agree in the following—

1. They first affect the anterior extremities.
2. They diminish respiration.
3. They produce hyperæsthesia during the latter stage of the poisoning process.

They differ in the following—

1. Theine principally influences sensation, while caffeine does not.
2. Theine produces spontaneous spasms and convulsions, while caffeine does not.
3. Theine impairs the nasal reflex early in the poisoning process, while caffeine does not, if at all, until in the very last stage.
4. The lethal dose of theine is larger than that of caffeine.—*Therapeutic Gazette.*

**Antifebrine,** the chemical name of which is acetanilide, has recently been introduced into therapeutics as a febrifuge. It is a crystalized substance, whitish, little soluble in water, but easily so in alcohol. It has scarcely an appreciable taste. Dr. Dujardin Beaumetz, who reported on this new medicament at a recent meeting of the Societe de Therapeutique of Paris, represented it as an antithermic in doses of from 25 to 50 centigrammes; it lowers the temperature, and provokes abundant perspiration. In larger doses, like carbolic acid, it determines cyanosis. In phthisical subjects it has not produced any favorable results. The members present almost unanimously condemned antifebrine as a dangerous antipyretic, and preferred having recourse to antyprine, which, at least, does not offer any danger, provided it is not administered in excessive doses. Dr. G. R. Elliott, of New York, has used this substance successfully in typhoid fever.—*Eastern Med. Journal.*

**Acute Rheumatism in Philadelphia Hospitals.**—Dr. J. C. Wilson has, during the past three years, used in acute rheumatism a mixed treatment, consisting of sodium salicylate in combination with sodium bicarbonate or sodium acetate. The formula commonly employed is as follows:

℞. Sodii salicylatis............................gr. xv,
Sodii bicarbonatis..........................gr. xxx,
Aqua menth. pip............................℥ ss. M.
To be taken every third or fourth hour.

This dose is increased or diminished in accordance with the intensity of the pyrexia, the acuteness of the joint pains and the condition of the heart, in individual cases. After the reaction of the urine becomes distinctly alkaline under this treatment, the sodium bicarbonate is stopped and the salicylate continued alone at gradually increasing intervals until the pains and fever have wholly ceased. The administration of iron then follows, usually in the form of Basham's mixture, the mistura ferri et ammonii acetatis, U. S. P.

Dr. Wilson regards this method as quite equal to the treatment by salicylic acid, the sodium salicylate alone or by salicin, as regards the rapidity with which the pain is relieved and the fever controlled, and as much superior to them in the diminished danger of producing the toxic effects of large doses of the salicylates, especially cardiac depressions, epistaxis, tinnitus, headache and maniacal excitement—phenomena which are very seldom observed, if we except slight ringing in the ears, under this plan of treatment.

The diet consists chiefly of milk; light broths are, however, occasionally permitted, to which. with beginning convalescence, are added bread, rice, custards, etc. Alcohol in any form or dose is scarcely ever given, except in emergencies, and forms no part of the treatment even during the convalescence.—*North Carolina Medical Journal.*

**How Long to Wait Before Applying the Forceps.**—Dr. L. Ch. Boisliniere, of St. Louis, tells us in the *New England Med. Monthly:* If the head is engaged at the superior strait in a moderately contracted pelvis, the condition of the woman good, the pains sufficient, and no accident threatens her life or that of her child, wait six to eight hours, in order to give sufficient time for the moulding of the head.

Frequently auscultate the child's heart. So long as its beats are strong and regular, there is no danger. If the beats of the heart become feeble, and especially if they intermit, apply the forceps at once.

When the head, in the excavation, has made no progress for one hour, deliver it with the forceps; for in this case, the pressure on the mother's soft parts may prove disastrous, and the compression of the child's head may produce such changes in its cerebral circulation as to cause its death before or soon after delivery.

If the labor becomes complicated by any serious accident, the forceps should be immediately applied.

He then proceeds to give some very valuable *maxims*, beginning with the statement that so long as the phenomena of labor are normal, and no accident threatens the life of the mother or the child, the duty of the obstetrician is patience, and he must know how to wait.

Interfere when there is a special indication; never otherwise. But as soon as the indication is present, act without temporizing; for timid midwifery is as bad as "meddlesome" midwifery.

In all obstetric operations, perseverance, slowness, groping even, whilst acting, must always precede the use of force.

When midwifery ceases to be expectant and becomes aggressive, action should be firm, intelligent, but never violent.

Avoid fuss and haste; never hurry, especially if you have only a little time left to operate.

Before proceeding to perform an obstetric operation, remove all sources of error. Verify the presentation, position of child, condition of the mother's pelvis and soft parts, for mistakes may have disastrous consequences. If, in your presentation, the usual methods of exploration fail, chloroform your patient to the surgical extent, and use you whole hand in the vagina; for an exact knowledge of the presentation is absolutely required before acting. remember the possibility of rupturing the vagina, if it be narrow, and proceed very slowly, gently but firmly.

Finally, every obstetric operation is a heroic measure; the success of it depending upon the favorable moment when it is undertaken, upon the method selected and the skill of the operator.

**Antifebrine.**—This new antipyretic has been used by Dr. Geo. R. Elliot, of New York, in one case of typhoid fever with better results than those obtained from any other antipyretic agent. Four grains were administered at 4 p. m., 7 p. m., and 10 p. m., respectively, on the the twelfth day of the disease, with the following results: The temperature at the time of giving the first dose was 104½°, pulse 115; half an hour after giving the second dose the patient began to perspire profusely, and at eight o'clock the temperature had fallen to 102½°. The pulse had not varied in frequency, but its tension had slightly increased. The reduction of the temperature was attended by an amelioration of the symptoms. The mind became clear and the patient conversed rationally. At ten o'clock the temperature had reached 104°, and another dose of 4 grains was given with a repetition of what followed the second dose—viz: the temperature falling to 102°, accompanied by copious perspiration, and no perceptible bad effect upon the heart. At 3.30 a. m. the temperature had reached 103 2-5°. Four grains of antifebrine were given, and at the end of thirty minutes there was profuse perspiration, accompanied by a fall of temperature which at 5.30 a. m. registered 101 4-5°. A morning remission being established on the seventeenth day of the disease, the antifebrine was discontinued. At no time was it found necessary to give more than 20 grains during twenty-four hours. Stimulants were used spairingly. While the antifebrine was a success in lowering the temperature, the patient succumbed to perforation of the intestine five days after the antifebrine was discontinued.—*Practice.*

**Transmission of Symptoms of Hysteria under the Influence of a Magnet.**—M. Babinsky, in a paper read at the Paris Biological Society, stated that, under the influence of a magnet, certain hysterical symptoms may be transferred from one person to another, even should these persons be placed at some distance from each other. In a first series of experiments, practiced upon two hystero-epileptic patients capable of being hypnotized, the hemianæsthesia from which they suffered was transmitted from one to the other, as well as from other symptoms produced in one of the patients by suggestion; different forms of paralysis, brachial or crural monoplegia, hemiplegia, paraplegia, coxalgia, dumbness, etc. In a second series of experiments, one of the above mentioned patients was placed in communication with patients suffering from different forms of hysterical spontaneous paralysis. These symptoms were transmitted to the hypnotized patient, but continued in the patients who exhibited them. After two consecutive experiments an improvement was observed in one case of spontaneous paralysis. In a case of hemiplegia following an hysterical attack, M. Babinsky succeeded in causing the disappearance of paralysis after four consecutive experiments, such as above described. A method of treatment is thus determined. The author has proved by other experiments that hysterical symptoms are not the only ones capable of transmission. He succeeded in transmitting other symptoms, such as paralysis, tremor, etc., combined with organic changes of the nervous system. In these experiments, made in Dr. Charcot's ward at the Saltpetriere, suggestions or simulation was carefully avoided.—*Medical News.*

**Typhoid from a Single Dose.**—M. Dujardin Beaumetz has forwarded to the Paris Academy of Sciences a communication on the Pierrefonds typhoid cases last summer. M. Fernet, who occupies a high post at the Ministry of Public Instruction, his wife and family, hired a house at Pierrefonds, a fashionable resort near Compiegne, contiguous to two others. After they had rented it for the season they were told to beware of the water in the well. On this account they drank exclusively mineral water until the last day, when the stock was out, and the servants were too busy preparing to return to Paris to go to fetch some bottles from the chemist. Madame Fernet said, "For once surely there can be no harm in drinking the well-water." They drank it. Six out of the nine persons have since died, including one of the servants. The cook, two of the four children, and Madame Fernet had had typhoid fever before, and though attacked again by it after their return from Pierrefond-, have got through the illness. The well has been examined and is reported to contain the bacilli which are believed to be associated with typhoid fever.

**Antiseptic Treatment of Croup.**—The following plan of treatment is recommended by M. Renon, (*Journal de Medical de Paris*): The patient is placed in a well ventilated room of medium size, the temperature of which is maintained at from 68° to 75° F. Upon an oil stove is kept a vessel of a capacity of two quarts, in which the water is constantly boiling. Into this is put every three hours a tablespoonful of a mixture of salicylic acid, 56 parts; benzoic acid, 112 parts; carbolic acid, 280 parts, and alcohol, 468 parts. The stove is placed near the bed and the steam impregnated with this mixture is conducted by means of suitably arranged curtains, to the patient. The patient is kept in this atmosphere until the symptoms have entirely disappeared, and for two or three days thereafter, and if tracheotomy has been performed, until the wound is closed. A close watch should be maintained over the case, and if any symptoms of poisoning are manifested the quantity of carbolic acid should be diminished.—*Buffalo Medical and Surgical Jonrnal.*

**Buttermilk in Sick Stomach.**—An irritable stomach, it will be admitted, is often a most serious complication in the management of sickness. In occasional cases, of no particular gravity otherwise, oftenest in disease of children, this difficulty leads to a fatal issue. Buttermilk, so far as I am aware, is an untried remedy in such cases. I have had some experience recently with it, quite satisfactory in a few instances. For cases of persistent vomiting occuring in succession, intolerant of any other treatment, gave way kindly to this. * * * So far as I have observed, buttermilk does not coagulate in the stomach, as does new milk. This is, perhaps, its only advantage over the latter, but one of inestimable value, since the coagulation of new milk casein, so likely to occur, utterly forbids its use in many cases. In the summer "complaints" of children, for instance, buttermilk might be found eminently appropriate.—*Therapeutic Gazette.*

**Mammary Abscess,** according to the experience of Drs. Elliot, Murphy and Busey, of Washington, D. C., can be very sccessfully treated by the local application of the spirits of turpentine. The former gentleman so far believes in the abortive power of this agent in the condition mentioned, that he always resorts to it before testing the general methods laid down, such as strapping, application of belladonna, the ice-bag, etc. After bathing the breast well with turpentine, a piece of cotton flannel wrung out of turpentine is applied and allowed to remain—say half an hour. This is repeated three or four times daily. The breast is washed before the child is nursed. It is claimed that it is not necessary to stop nursing the infant from the affected breast.—*Practice.*

**A writer** in the *American Pharmacist* says: "While we must never be off our guard against accidents, yet we must exercise circumspection lest we offend our customers. It was but a few months since that a man came into my store and asked for two ounces of oxalic acid; while doing it up I asked him for what purpose he intended to use it, and received the polite reply, 'It is none of your business; I will pay you for all I buy.' I replied that 'while I did not intend to pry into his affairs, I always make a practice of asking the question when I sold poison.' 'Poison!' cried he, 'is that poison?' 'Yes sir, it is.' 'Why, my wife wants it to put in pies.' I gave him tartaric acid, and explained his mistake."—*Druggist.*

**Coniin Hydrobromate.**—Coniin, from conium maculatum. It has been used in a variety of diseases. It is now used in spasmodic affections. The crystallizable salts—the hydrobromate—is a substitute for pure conium on account of its easier administration and uniform composition. Dose, 1-12th grain.—*Ibid.*

**Cannabin Tannate.**—This glucoside, combined with tannic acid, was introduced by Merck. It acts as a pleasant hypnotic, and is used in cases where morphia is objectionable. The dose is 1 centigramme.—*Ibid.*

**A Cure for Warts.**—It is now fairly established, says a writer in the *Medical Press*, that the common wart, which is so unsightly and often so proliferous on the hands and face, can be easily removed by small doses of sulphate of magnesia taken internally. M. Colrat, of Lyons, has drawn attention to this extraordinary fact. Several children treated with 3-grain doses of Epsom salts, morning and evening, were promptly cured. M. Aubert cites the case of a woman whose face was disfigured by these excrescences, and who was cured in a month by a drachm and a half of magnesia taken daily. Another medical man reports a case which disappeared in a fortnight from the daily administration of 10 grains of the salts.—*Northwestern Lancet.*

**Arsenite of Strychnine** is recommended by Roussell as a substitute for arsenical preparations, especially Fowler's solution. Fowler's solution has its disadvantages inasmuch as patients readily become accustomed to it, and when given in large doses it is apt to produce intoxication. When injected subcutaneously it does not possess the above mentioned disadvantages, but has little effect. Arsenite of strychnine injected hypodermically produces excellent results and is not painful. The dose to begin with should be small (0.001 gm). Arsenite of strychnine is a powerful antiseptic. In abdominal typhus it produces excellent results. Combined with salicylate of iron it is given in chronic anæmia, dyspepsia, etc.—*Journal of Pharmacy.*

**Adonidin.**—The amorphous bitter principle of *adonis vernalis*, slightly soluble in ether and water but much more so in alcohol. Its effects are marked in increasing the amount of urine. It diminishes the heart beats and increases its strength. It rapidly raises vascular tension, the heart's action becoming stronger and more regular under its influence. It has no special action on nervous troubles of the heart. It has been used in cases where digitalis is indicated, and is said to be less dangerous. The dose is from 5 to 8 grains.—*American Journal.*

**Antifebrin.**—Is a white crystalline powder possessing neither basic or acid properties and non-poisonous. It is odorless and colorless; insoluble in cold water, slightly soluble in boiling water, freely soluble in ether and alcohol, and possesses antipyretic properties. It is destined to supersede antipyrin and thallin. The dose is from 4 to 16 grains.—*American Medical Journal.*

**Scoparin and Spartein.**—From broom tops. Scoparin in stellate crystals. Used as a diuretic in doses of three centigrammes, hypodermically. Spartein is a narcotic alkaloid, used as a diuretic. Given in doses of 4 centigrammes.—*Ibid.*

**To Remove Stains of Iodine from the Skin.**—The sulphide of sodium, or sodium sulphydrate, in a 10 or 20-per cent. watery solution, applied upon a compress, will remove the stain of iodine, as well as allay its irritant action when excessive.—*Ex.*

